# Heat Shock Protein 90 Has Roles in Intracellular Calcium Homeostasis, Protein Tyrosine Phosphorylation Regulation, and Progesterone-Responsive Sperm Function in Human Sperm

**DOI:** 10.1371/journal.pone.0115841

**Published:** 2014-12-26

**Authors:** Kun Li, Yamei Xue, Aijun Chen, Youfang Jiang, Haifeng Xie, Qixian Shi, Songying Zhang, Ya Ni

**Affiliations:** 1 Department of Reproductive Physiology, Zhejiang Academy of Medical Sciences, Hangzhou, Zhejiang 310013, China; 2 Reproductive Medicine Center, Department of Obstetrics and Gynecology, Sir Run Run Shaw Hospital, College of Medicine, Zhejiang University, Hangzhou, Zhejiang 310016, China; China Agricultural University, China

## Abstract

Heat shock protein 90 plays critical roles in client protein maturation, signal transduction, protein folding and degradation, and morphological evolution; however, its function in human sperm is not fully understood. Therefore, our objective in this study was to elucidate the mechanism by which heat shock protein 90 exerts its effects on human sperm function. By performing indirect immunofluorescence staining, we found that heat shock protein 90 was localized primarily in the neck, midpiece, and tail regions of human sperm, and that its expression increased with increasing incubation time under capacitation conditions. Geldanamycin, a specific inhibitor of heat shock protein 90, was shown to inhibit this increase in heat shock protein 90 expression in western blotting analyses. Using a multifunctional microplate reader to examine Fluo-3 AM-loaded sperm, we observed for the first time that inhibition of heat shock protein 90 by using geldanamycin significantly decreased intracellular calcium concentrations during capacitation. Moreover, western blot analysis showed that geldanamycin enhanced tyrosine phosphorylation of several proteins, including heat shock protein 90, in a dose-dependent manner. The effects of geldanamycin on human sperm function in the absence or presence of progesterone was evaluated by performing chlortetracycline staining and by using a computer-assisted sperm analyzer. We found that geldanamycin alone did not affect sperm capacitation, hyperactivation, and motility, but did so in the presence of progesterone. Taken together, these data suggest that heat shock protein 90, which increases in expression in human sperm during capacitation, has roles in intracellular calcium homeostasis, protein tyrosine phosphorylation regulation, and progesterone-stimulated sperm function. In this study, we provide new insights into the roles of heat shock protein 90 in sperm function.

## Introduction

Heat shock protein 90 (Hsp90), a highly conserved molecular chaperone [1], plays critical roles in client protein maturation, including that of kinases and transcription factors, as well as in signal transduction, protein folding and degradation, and morphological evolution [2]. Hsp90 has been studied in the sperm of diverse mammals including rats, mice, boars, stallions, dogs, cats, rabbits, rhesus macaques [3], and humans. Hsp90 is expressed in the testis during rat development [4] and interacts with Hsp70 in testicular cells [5]. Hsp90 has ATPase activity that is stimulated by nuclear autoantigenic sperm protein in the nuclei of mouse sperm [6]. The absence of Hsp90a in mice results in meiotic arrest towards the end of the pachytene stage, resulting in germ cell loss and reduction in testicular size [7]. Sperm deficient in Hsp90b1 exhibit large and globular heads and abnormal midpieces, and are unable to fertilize oocytes [8]. In boar ejaculate, Hsp90AA1 protein levels vary significantly depending on the temperature at which it is cryopreserved [9]. Geldanamycin (Geld), an inhibitor that specifically blocks Hsp90 function by competing for ATP binding [13], decreases porcine sperm motility [10], [11]. Cryopreservation decreases Hsp90 levels and motility in human sperm [12], and Hsp90 is tyrosine phosphorylated during capacitation [13].

Capacitation, the process by which mammalian sperm becomes competent for fertilization, occurs only after sperm has resided in the female genital tract for a certain period of time [14], [15]. During capacitation, many biochemical and physiological changes occur in sperm, including alterations in the cholesterol content, plasma membrane phospholipid composition, intracellular ion flux, and protein tyrosine phosphorylation [16]. Capacitated sperm usually exhibit hyperactivation, which is regulated by a number of Ca^2+^ channels and pumps [17]. In addition, sperm capacitation is associated with protein tyrosine phosphorylation that occurs via protein kinase A and cSrc family kinase signaling pathways [16]. Capacitated sperm acquire the ability to bind specifically to the zona pellucida [18] of oocytes, which initiates the acrosome reaction (AR) [19], [20], the terminal event in the acquisition of fertilizing ability [15]. Progesterone (P4) is present in the follicular fluid surrounding the oocyte as well as in the female reproductive tract at micromolar concentrations [21], [22]. P4 stimulates sperm hyperactivation, motility, capacitation, chemotaxis, and AR, processes that are mediated by calcium influx, protein tyrosine phosphorylation, and other signaling cascades [23]. However, it remains unclear how Hsp90 exerts its actions in human sperm.

Therefore, our goal with this study was to elucidate how Hsp90 affects intracellular calcium homeostasis, protein tyrosine phosphorylation, capacitation, hyperactivation, and motility in sperm in response to progesterone. We provide novel insights into the roles of Hsp90 in sperm function.

## Materials and Methods

### Reagents

Human tubal fluid (HTF) medium was used as described previously [24]–[26]. Percoll was purchased from Pharmacia LKB (Uppsala, Sweden). P4 was obtained from ICN Biomedicals (Irvine, CA, USA). Dimethyl sulfoxide (DMSO) was acquired from Merck (Darmstadt, Germany). Geld was provided from Cell Signaling Technology (Danvers, MA, USA). Hoechst 33258 was purchased from Sigma-Aldrich (St. Louis, MO, USA). Antibodies used in this study are as follows: rabbit polyclonal anti-Hsp90 antibody (ab13495; Abcam Co., Hong Kong), β-tubulin antibody (ab6046; Abcam Inc., Cambridge, MA, USA), rabbit IgG (R&D Systems, Minneapolis, MN, USA), Alexa Fluor 555-conjugated goat anti-rabbit antibody, peroxidase-conjugated goat anti-rabbit IgG (H+L) (Molecular Probes, Invitrogen, Carlsbad, CA, USA), and anti-phosphotyrosine monoclonal antibody (clone PY20; Invitrogen, Camarillo, CA, USA).

### Semen collection and sperm preparation

This study was approved by the Medical Ethics Committee at Zhejiang Academy of Medical Sciences. Written informed consent was obtained from all donors (aged 25–35) prior to specimen collection. Forty-one semen samples were collected by masturbation from healthy men after 3–5 days of abstinence. Each sample, collected in a sterile container, was allowed to liquefy for 45 min at 37°C before being processed. The routine analysis of all samples was as set out by the World Health Organization [27]. Only samples that met the following criteria were used in the study: motility greater than or equal to 50% (a+b grade), viability greater than or equal to 85%, sperm concentrations greater than 20×10^6^ sperm/ml, and the percentage of normal sperm morphology greater than or equal to 15%.

Sperm preparation was performed as previously described [25], [26]. Briefly, sperm were separated from seminal plasma by centrifugation at 600×*g* for 15 min through a 40% and 80% Percoll discontinuous gradient containing 4 mg/ml bovine serum albumin. Approximately 200-µl pellets were washed twice in centrifuge tubes with 2 ml of fresh HTF media by performing centrifugation at 500×*g* for 5 min. Sperm concentrations were then adjusted to 20×10^6^ cells/ml and incubated at 37°C in 5% CO_2_ for 3 h. During the incubation period, aliquots were treated with the indicated reagents, according to the experimental design.

### Indirect immunofluorescence staining

Sperm were washed twice in phosphate-buffered saline (PBS), fixed in 4% paraformaldehyde for 15 min, and mounted on Silane-Prep slides (Sigma-Aldrich). Cells were air dried, permeabilized with 0.1% Triton X-100 in PBS for 10 min, and washed with PBS at room temperature. Nonspecific reactive sites were blocked for 1 h with 10% goat serum at room temperature. The cells were then incubated overnight at 4°C with a rabbit Hsp90 antibody (1∶250) or with normal rabbit IgG used as a negative control. After being washed three times with PBS at 10-min intervals, cells were incubated with Alexa Fluor 555-conjugated anti-rabbit IgG secondary antibody (1∶400) for 1 h at 37°C. Following incubation with Hoechst 33258 and washes in PBS, cells were examined by fluorescence microscopy (Nikon Eclipse 80i; Nikon Inc., Tokyo, Japan).

### Measurement of intracellular calcium ([Ca^2+^]_i_) levels in human sperm

The [Ca^2+^]_i_ levels in sperm were measured using Fluo-3 AM according to an adapted method described previously [28]. In brief, prepared sperm were loaded with the fluorescent calcium probe Fluo-3 AM (10 µM) in the dark at 37°C for 30 min and were then washed twice with HTF and centrifugation at 300×*g* for 5 min to remove free Fluo-3 AM. Fluo-3 AM-loaded sperm were resuspended and incubated at 37°C for 20 min. Fluo-3 AM-loaded sperm aliquots (10^6^ cells/ml) were exposed to the vehicle control (0.3% DMSO in HTF, v/v) or 20 µM Geld in the absence or presence of 2.5 µM P4. The time course of [Ca^2+^]_i_ fluorescence signals was then monitored using the Synergy 2 Multi-Function Microplate Reader (Bio-Tek Instruments, Winooski, VT, USA), at 480/20-nm excitation and 520/20-nm emission wavelengths. After fluorescence analysis, sperm were confirmed to be motile by using microscopy. The fluorescence intensity of Fluo-3 was recorded for 30 min at 3-min intervals. First recorded raw intensity values were set as 100% and were used to normalize the other raw intensity values.

### Protein extraction and western blot analysis

Sperm were washed twice with PBS and protein was extracted in sodium dodecyl sulfate (SDS) lysis buffer (P0013G; Beyotime Institution of Biotechnology, Haimen, China) containing Roche Complete Mini EDTA-free Protease Inhibitor Cocktail, PhosSTOP phosphatase inhibitors, and 1 mM phenylmethylsulfonyl fluoride added immediately before usage. The concentrations of sperm protein extracts were quantified using the Bicinchoninic Acid Assay Kit (Beyotime Institution of Biotechnology, Haimen, China). Next, protein extract aliquots were mixed with loading buffer and boiled for 5 min. Equal amounts of protein were subsequently resolved by SDS-polyacrylamide gel electrophoresis (PAGE) on 10% acrylamide gels and transferred to Immunoblot-P membranes (Millipore Corporation, Bedford, MA, USA). After being blocked with 5% non-fat milk in Tris-buffered saline (TBS; pH 7.4) for 2 h at room temperature, membranes were incubated with mouse anti-phosphotyrosine antibody (clone PY-20; 1∶1,000) at 4°C overnight, followed by 3 washes (5 min each) with TBS containing 0.01% (v/v) Tween-20. The membranes were then incubated with peroxidase-conjugated mouse IgG (1∶5,000) at room temperature for 1 h and washed three times. Protein bands were detected using the Enhanced Chemiluminescence Kit (Pierce Biotechnology, Rockford, IL, USA), according to the manufacturer's instructions. When required, membranes were stripped for immunoblotting with β-tubulin antibody (1 µg/ml). Molecular weights of detected proteins were deduced by comparison with the prestained protein ladder (Fermentas, Thermo Fisher Scientific Inc., Burlington, NC, USA). The gray intensity was quantified using Image J software.

### Assessment of sperm capacitation

Capacitation was evaluated indirectly by P4-induced AR, based on the premise that only capacitated sperm undergo exocytosis [29], [30]. The AR was assessed by chlortetracycline (CTC) staining as previously described [24], [25], [31]. Prepared sperm were exposed to 0.25–2.0 µM Geld in HTF, either in absence or presence of 2.5 µM P4 for 3 h. Sperm were washed twice to remove Geld and/or P4, and were then induced by 15 µM P4 for 15 min prior to evaluation of the AR. Samples were immediately fixed by mixing gently with an equal volume of 8% glutaraldehyde at 37°C for 5 min and were then stained with CTC at 37°C for 10 min. To distinguish the live from dead sperm, Hoechst 33258 (1 µg/ml final concentration) staining was performed for 2 min in the dark at 37°C. Samples were then immediately observed under a fluorescence microscope (Nikon 80i, Tokyo, Japan) at 1000× magnification, using a mercury excitation beam passing through a 380-nm filter and fluorescence emission through a 420-nm dichroic mirror. Only live Hoechst 33258-negative sperm were assessed. The three following patterns of CTC staining were observed: F pattern, fluorescence over the entire head including the equatorial region, indicating non-capacitated, acrosome-intact sperm; B pattern, fluorescence-free band in the postacrosomal region, representing capacitated acrosome-intact sperm; and AR pattern, very weak fluorescence over the head, corresponding to sperm that underwent AR. At least 200 cells from each sample were assessed by two independent researchers.

### Sperm motility and hyperactivation analyses

Sperm motility and hyperactivation were analyzed using a computer-assisted sperm analyzer (CASA; IVOS; Hamilton-Thorne Bio-sciences, Beverly, MA, USA), as described previously [32]. The CASA parameters were set up as follows: frame rate, 60 Hz; acquisition frame, 30; minimum contrast, 80; minimum cell size, 3 pixels; cell intensity, 40; path velocity, 25.0 µm/s; straightness threshold, 80%; slow cell, average path velocity (VAP) and straight line velocity (VSL) of less than 5.0 µm/s and 11 µm/s, respectively; illumination intensity, 2,164; magnification, 1.73; temperature, 37°C; and chamber depth, 20 µm. For each sample, at least 200 motile sperm were examined. Aliquots of sperm (2 µl) were loaded into 20-µm-deep chambers of Leja slides warmed to 37°C. The following sperm parameters were assessed: VSL, VAP, curvilinear velocity (VCL), straightness (STR, VSL/VAP multiplied by 100), linearity (LIN, VSL/VCL multiplied by 100), amplitude of lateral head displacement (ALH), beat-cross frequency (BCF), and the percentage of motile, progressive, and hyperactivated sperm. Sperm hyperactivation was defined by the SORT function as follows: VCL, greater than or equal to 150 µm/s; ALH, greater than or equal to 7.0 µm; and LIN, less than or equal to 50% [32], [33].

### Statistical analysis

Results are expressed as the means ± standard error of the mean (SEM). Statistical analyses were performed by one-way analysis of variance (ANOVA) using SPSS 13.0 software. When the test of homogeneity of variances was significant (P<0.05), data were analyzed by the Dunnett's T3 test; otherwise, the LSD test was used. P<0.05 was considered statistically significant.

## Results

### Hsp90 expression in human sperm increases during capacitation

**Figure 1:**
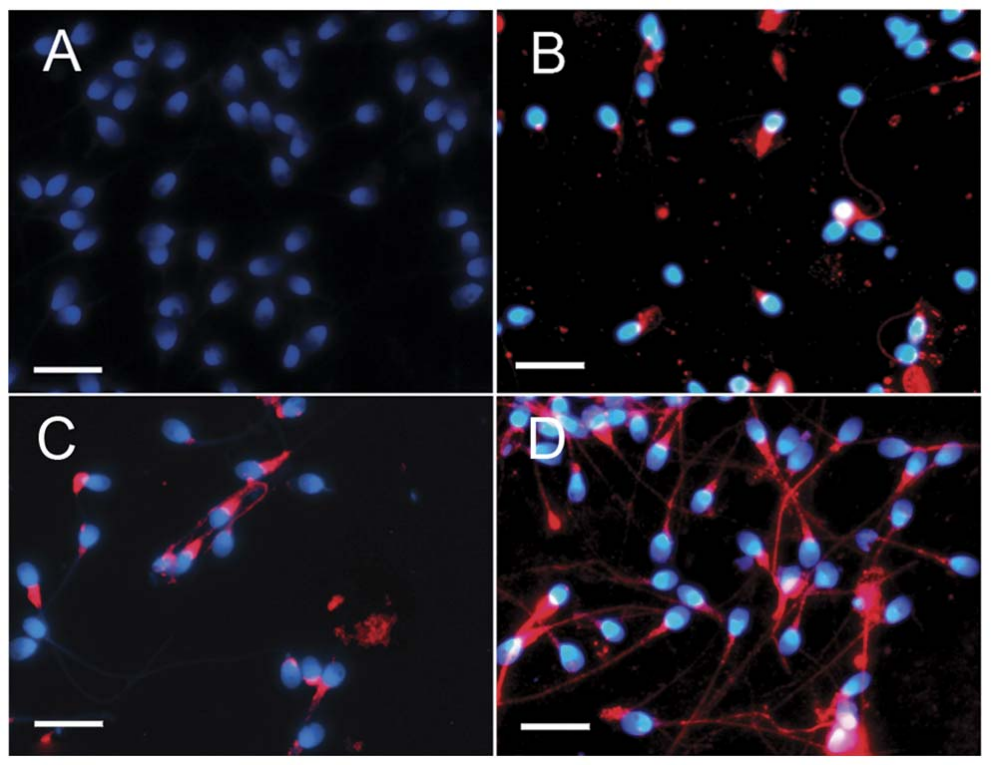
Hsp90 expression in human sperm under various conditions. Sperm were stained with Hsp90 antibody (red) or rabbit IgG (control). Hoechst 33258 staining (blue) marks cell nuclei. A) Negative control, rabbit IgG used as the primary antibody. B) Sperm not subjected to Percoll isolation. C) Sperm isolated using Percoll isolation (0 h) D) Sperm isolated using Percoll under capacitation conditions (3 h). Bar  = 10 µm. Experiments were performed using specimens collected from five different men.

**Figure 2:**
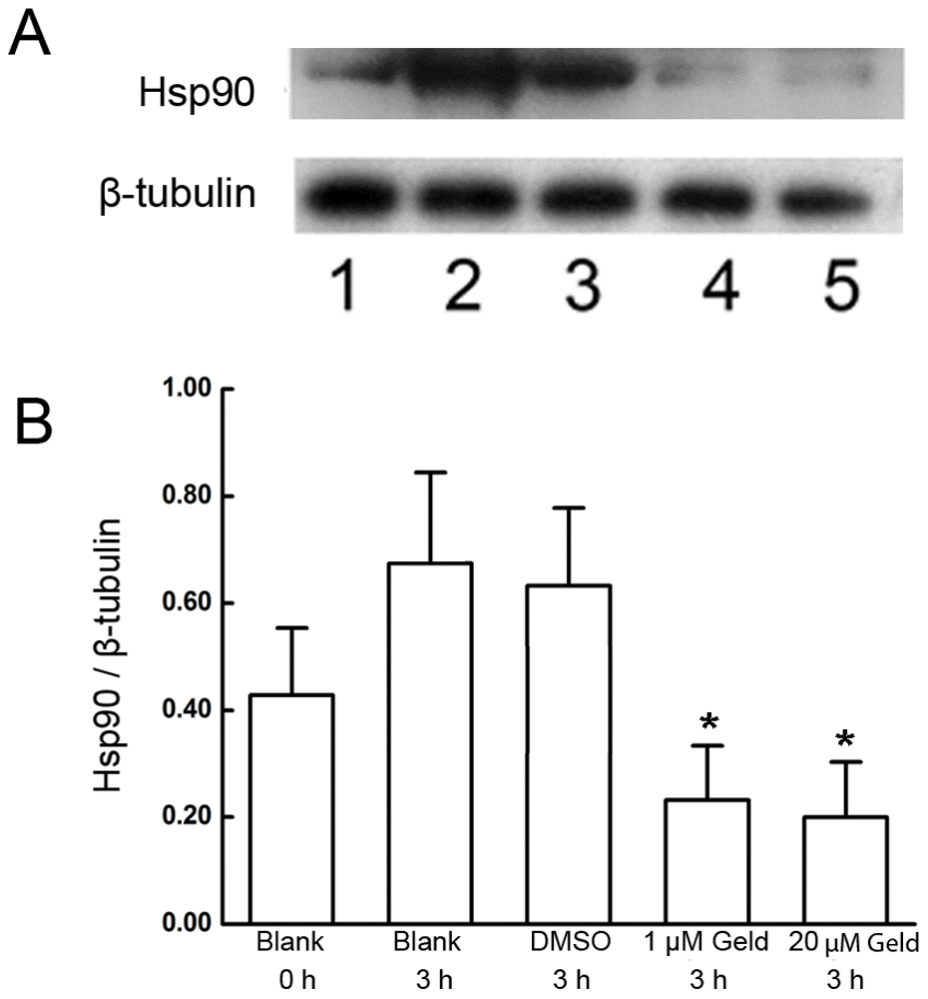
Effect of Geld on Hsp90 expression during capacitation. A) Under capacitation conditions, sperm were incubated in absence of geldanamycin (Geld) as a blank control for 0 h (lane 1) and 3 h (lane 2); with dimethyl sulfoxide (DMSO) as the vehicle control (lane 3), 1 µM Geld (lane 4), and 20 µM Geld (lane 5) for 3 h. Protein concentrations of sperm lysates were quantified using the Bicinchoninic Acid Assay Kit. Equal amounts of protein were resolved by SDS-PAGE, transferred to Immunoblot-P membrane, and probed with Hsp90 antibody. B) Band intensity ratios of Hsp90 to β-tubulin were lower in the Geld-treated sperm compared with that treated with the DMSO vehicle control, *P<0.05 (four independent experiments using ten different semen samples). Band intensities were measured using Image J software.

Changes in Hsp90 expression during capacitation were assessed using indirect immunofluorescence staining to investigate the biological significance of Hsp90. We found that Hsp90 was expressed predominantly in the neck, midpiece, and tail regions of human sperm (Fig. 1 B) and markedly increased in expression during capacitation (Fig. 1 C, D). Increase in Hsp90 expression during capacitation was well confirmed by the results of western blotting (Fig. 2 A, lanes 1, 2). In sperm isolated using Percoll and capacitated for 3 h, Hsp90 localization was not changed (Fig. 1 C, D). When sperm were cultured under capacitation conditions for 3 h (Fig. 1 D), the fluorescence intensity in the tail was markedly increased, suggesting that Hsp90 expression had increased. When the rabbit IgG was used as the negative control in place of the primary antibody, no fluorescence signal was observed (Fig. 1 A), indicating the Hsp90 antibody is specific.

### Geld inhibits the increase of Hsp90 expression during capacitation

Whether Hsp90 expression during capacitation is regulated by Geld is not known. To examine the effect of Geld treatment on Hsp90 expression during capacitation, western blotting was performed. The results showed that Geld decreased Hsp90 expression in a dose-dependent manner. Based on preliminary experiments, sperm were exposed to 1 µM and 20 µM Geld for 3 h. By comparing Hsp90 expression levels to that in the corresponding experimental control (Fig. 2 A, lanes 1, 2), we observed that the increase in Hsp90 expression that occurs during capacitation was blocked by a 3-h Geld treatment (Fig. 2 A, lanes 3, 4, and 5). The band intensity ratios of Hsp90 to β-tubulin from sperm treated with 1 µM and 20 µM Geld for 3 h were significantly lower when compared with that of sperm treated with the 0.1% DMSO control for 3 h (P<0.05, Fig. 2 B). These results indicate that the increase in Hsp90 expression during capacitation is inhibited by Geld.

### Geld blocks the elevation of [Ca^2+^]_i_ levels in human sperm during capacitation

**Figure 3:**
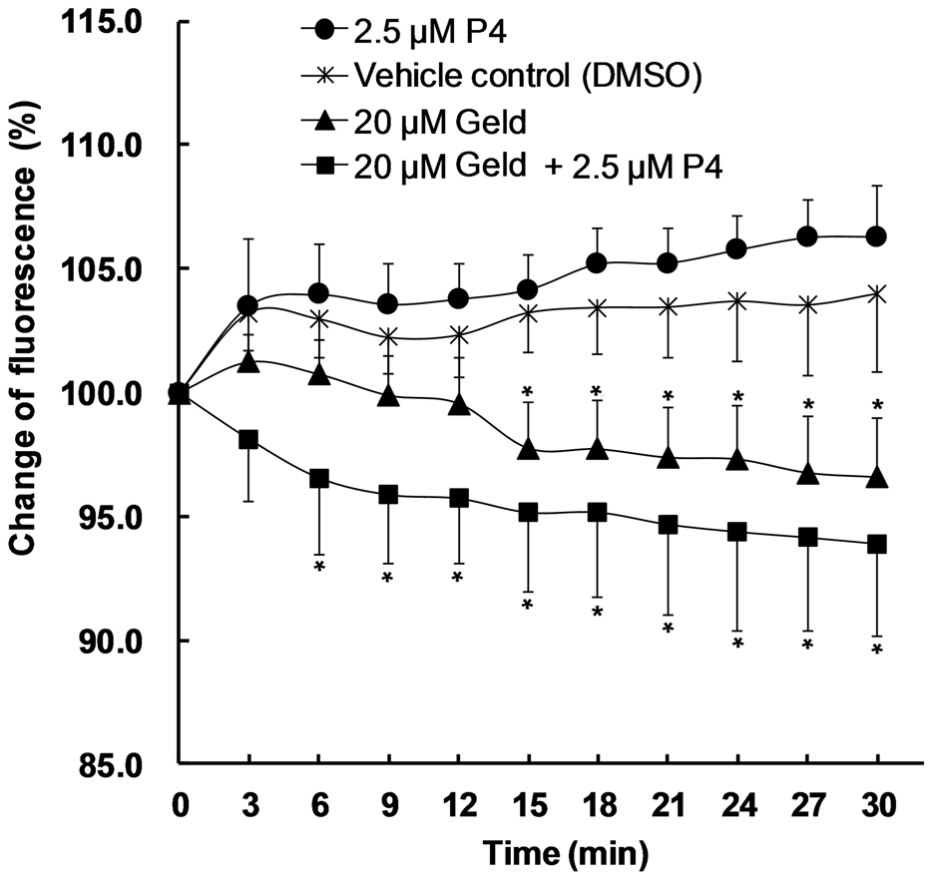
Effect of Geld on [Ca^2+^]_i_ in human sperm during capacitation. Sperm loaded with 10 µM Fluo-3 AM were washed twice with human tubal fluid (HTF) to remove free Fluo-3 AM. After incubation at 37°C for 20 min, Fluo-3 AM-loaded sperm aliquots (10^6^ cells/ml) were incubated with dimethyl sulfoxide (DMSO, 0.3%, v/v) as the vehicle control (star), 2.5 µM progesterone (P4, circle), 20 µM geldanamycin (Geld, triangle), or 20 µM Geld and 2.5 µM P4 (square). Treated sperm were then analyzed as described in Materials and Methods. The results are expressed as means ±SEM (n = 6); *P<0.05, compared to vehicle control.

To determine whether Hsp90 regulates Ca^2+^ signal in sperm, we examined the effect of Geld on [Ca^2+^]_i_ levels under capacitation conditions. Fluo-3 AM was loaded in sperm suspensions and the fluorescence intensities, indicating the [Ca^2+^]_I_ concentration, were measured using a microplate reader. As shown in Fig. 3, the fluorescence intensity increased substantially and persisted for 27 min in sperm exposed to the DMSO control or P4 alone. In contrast, Geld blocked and attenuated this increase in fluorescence intensity, as compared to the DMSO control (P<0.05). Upon exposure to 20 µM Geld and 2.5 µM P4, the fluorescence intensity lowered rapidly, when compared to that of cells treated with 20 µM Geld alone. These results suggest that Hsp90 regulates signaling cascades involved in calcium homeostasis.

### Geld enhances protein tyrosine phosphorylation in human sperm during capacitation

**Figure 4:**
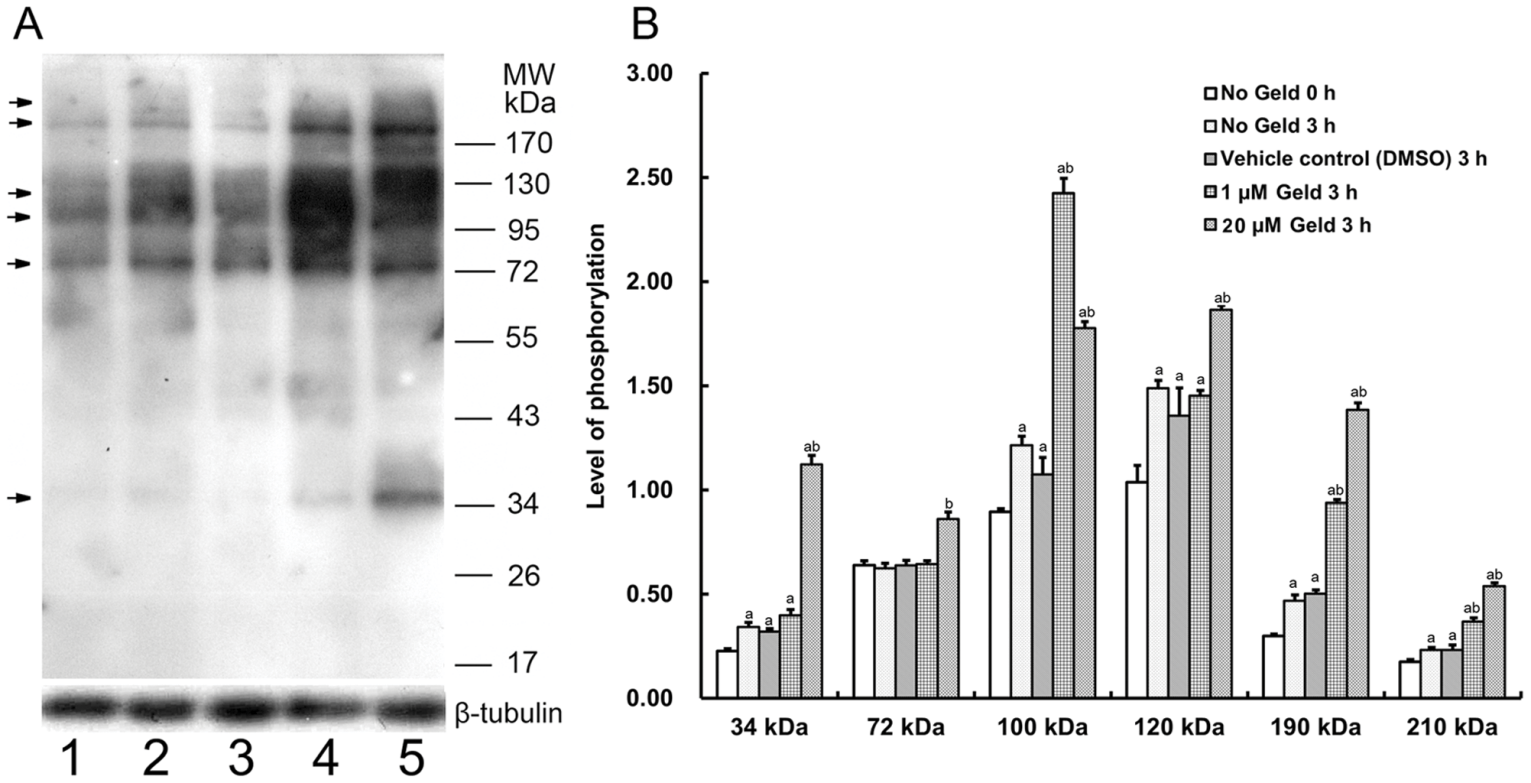
Effect of Geld on protein tyrosine phosphorylation in human sperm during capacitation. A) Sperm lysates were resolved by sodium dodecyl sulfate polyacrylamide gel electrophoresis (SDS-PAGE), transferred to Immunoblot-P membranes, and probed with anti-phosphotyrosine (PY20) antibody. Lanes 1, 2, 3, 4, and 5 contained lysates from sperm that were incubated in absence of geldanamycin (Geld) as blank control for 0 h and 3 h, with dimethyl sulfoxide (DMSO, 0.1%, v/v) as the vehicle control, 1 µM Geld, or 20 µM Geld for 3 h under capacitation conditions. B) The phosphorylation levels were quantified by the intensity ratio of bands to that of the loading control, β tubulin. The results are expressed as means ±SEM (n = 5). a, compared to the blank control at 0 h, P<0.05; b, compared to the vehicle control at 3 h, P<0.05.

The effect of Geld on tyrosine phosphorylation levels during capacitation was assessed by western blotting. Phosphorylation levels were expressed as the ratio of the experimental band intensity to that of the loading control β-tubulin (Fig. 4 B). Tyrosine phosphorylation of proteins including Hsp90 increased with increasing incubation times under capacitation conditions (Fig. 4 A, lanes 1 and 2). The addition of Geld at a concentration of either 1 µM or 20 µM significantly increased tyrosine phosphorylation of proteins with bands at 34, 72, 100, 120, 190, and 210 kDa (Fig. 4 A, lanes 4 and 5) compared to the DMSO control (Fig. 4 A, lane 3, P<0.05). The protein band at 34 kDa showed the most significant increase in tyrosine phosphorylation. These results suggest that Hsp90 is involved in regulating protein tyrosine phosphorylation during capacitation.

### Geld inhibits human sperm capacitation in response to P4

**Figure 5:**
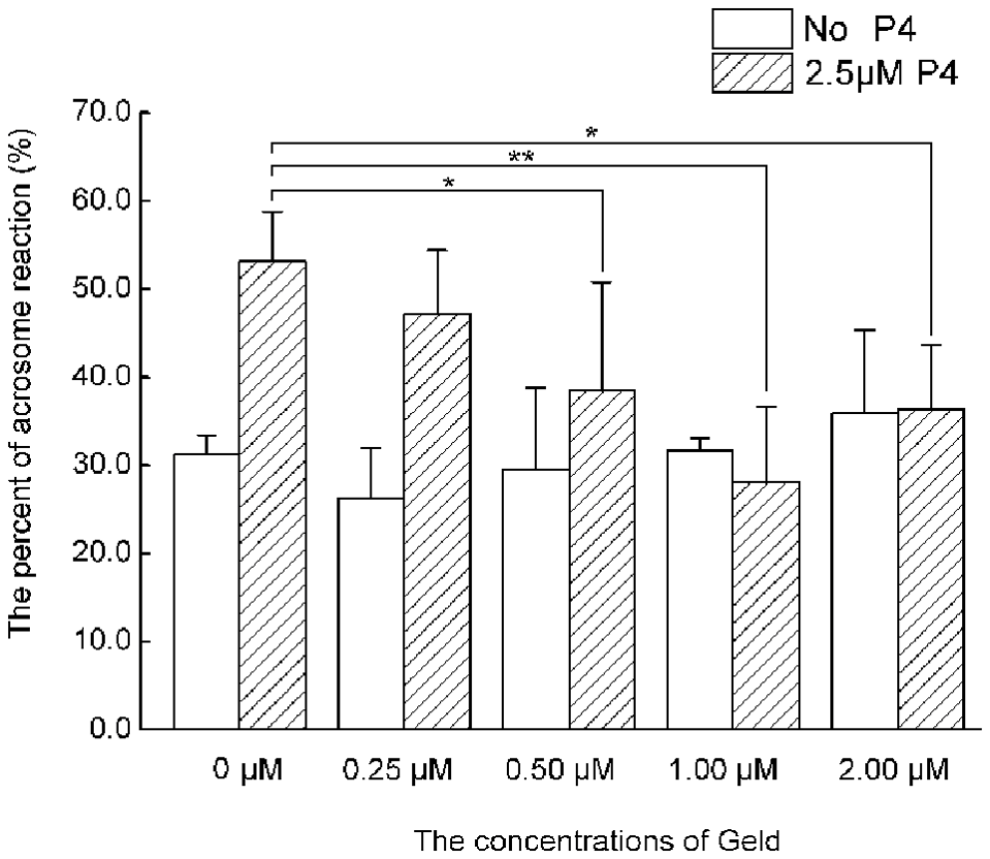
Effect of Geld on P4-induced capacitation in human sperm. The sperm were treated with various concentrations of geldanamycin (Geld) in presence or absence of 2.5 µM progesterone (P4) for 3 h under capacitation conditions. The acrosome reaction was evaluated by performing chlortetracycline (CTC) staining. Data represent means ±SEM (n = 6). In the presence of 2.5 µM P4, Geld treatment significantly reduced acrosome reaction compared to that in the vehicle control (0 µM Geld; *P<0.05 and **P<0.001). In the absence of P4, Geld treatment had no effect on the acrosome reaction compared to the vehicle control (0 µM Geld).

The effect of Geld on human sperm capacitation in the presence of P4 was evaluated by performing CTC staining. The AR served as an indicator of sperm capacitation. When P4 was absent during capacitation, no significant difference was observed in the AR between sperm treated with different concentrations of Geld (Fig. 5, P>0.05). Geld did not affect the AR, as compared to sperm exposed to the vehicle control. However, when 2.5 µM P4 was present, the P4-induced AR in response was attenuated by Geld in a dose-dependent manner (Fig. 5, P<0.05 or P<0.001), suggesting that Hsp90 is involved in human sperm capacitation in response to P4.

### Geld inhibits P4-induced hyperactivation and motility in human sperm

**Figure 6:**
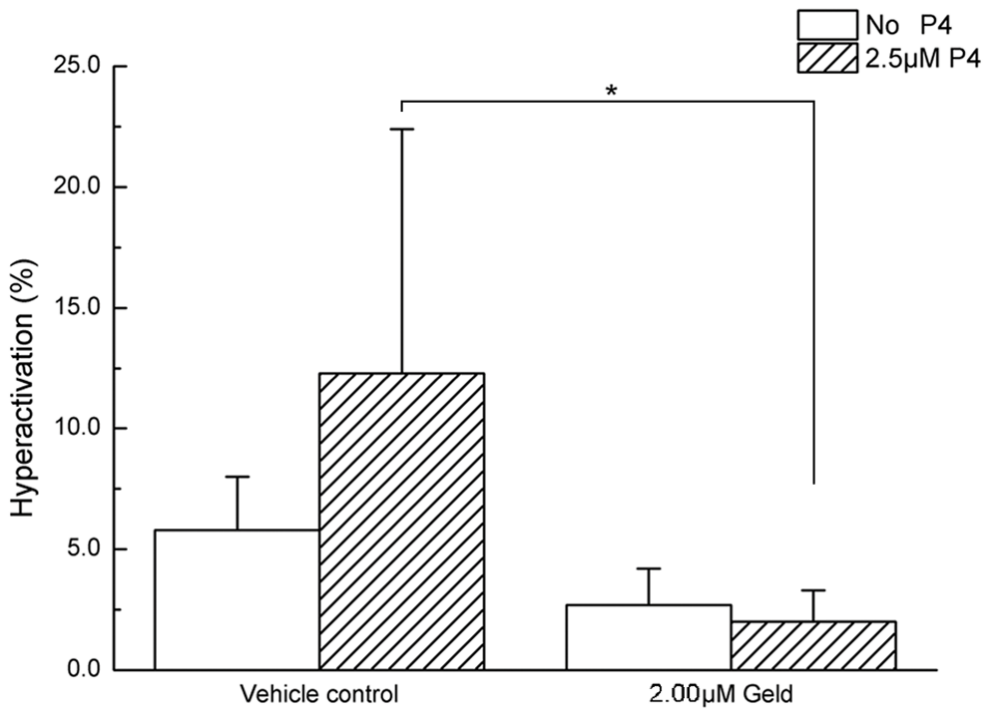
Effect of Geld on human sperm hyperactivation in response to P4. The sperm were treated with 2 µM geldanamycin (Geld) or with dimethyl sulfoxide (0.1%, v/v) as the vehicle control, in presence or absence of 2.5 µM progesterone (P4) for 3 h under capacitation conditions. Human sperm hyperactivation was analyzed using a computer-assisted sperm analyzer (CASA). Results are presented as means ±SEM (n = 6). In the presence of 2.5 µM P4, Geld treatment significantly reduced sperm hyperactivation, *P<0.05, 2.00 µM Geld vs. vehicle control. In the absence of P4, no difference between groups was observed.

The effects of Geld on hyperactivation and motility of human sperm were evaluated by CASA analysis. The results obtained were similar to those obtained when analyzing the effect of Geld on sperm capacitation. As shown in Fig. 6, no significant difference was observed in sperm hyperactivation between the vehicle control and 2 µM Geld treatment groups in the absence of P4 (P>0.05). In contrast, when 2.5 µM P4 was present, sperm hyperactivation was markedly decreased by 2 µM Geld, as compared to the control (P<0.05), suggesting that Hsp90 is involved in P4-induced sperm hyperactivation. The results of the analyzed sperm motion parameters are summarized in Table 1. In the absence of P4, sperm motion parameters were not significantly different between sperm treated with the vehicle control and 2 µM Geld (P>0.05). However, in the presence of 2.5 µM P4, sperm motion parameters including VSL, VAP, STR, and BCF, were attenuated significantly by 2 µM Geld treatment, as compared to the vehicle control (P<0.05), suggesting that Hsp90 has a role in P4-induced human sperm hyperactivation and motility.

**Table 1:** Effect of Geld on P4-induced motility parameters in human sperm.

	No P4		2.5 µM P4	
	Vehicle control	2.00 µM Geld	Vehicle control	2.00 µM Geld
VSL (µM/s)	56.9±2.3	49.5±3.5	62.4±2.6	45.2±3.5*
VCL (µM/s)	111.1±4.3	98.2±3.2	123.8±14.2	94.4±3.5
VAP (µM/s)	65.7±2.1	58.6±2.6	71.6±3.8	53.6±3.5*
STR (%)	83.5±1.0	82.3±2.2	84.3±0.9	61.0±1.2*
LIN (%)	50.0±1.6	49.7±1.8	50.3±3.7	46.7±1.5
ALH (µM)	5.2±0.2	5.1±0.3	5.7±0.8	5.1±0.1
BCF (Hz)	28.0±0.8	26.7±1.3	28.1±0.5	20.6±0.9 *
Progressive sperm (%)	41.3±3.4	38.7±±1.4	45.3 ± 1.7	31.0±7.9
Motility (%)	76.5±5.3	77.7±5.0	79.0±4.2	66.0±6.8*

Results are expressed as means ± standard error of the mean (SEM; n  =  6). In the presence of 2.5 µM progesterone (P4), geldanamycin (Geld) treatment significantly inhibited sperm motility, *P<0.05, 2.00 µM Geld vs. vehicle control. In the absence of P4, no difference between groups was observed. VSL, straight line velocity; VCL, curvilinear velocity; VAP, average path velocity; STR, straightness (VSL/VAP multiplied by 100); LIN, linearity (VSL/VCL multiplied by 100); ALH, amplitude of lateral head displacement; and BCF, beat-cross frequency.

## Discussion

To the best of our knowledge, we are the first to report that Hsp90 expression in human sperm increases during capacitation. The results showed that Hsp90 expression, which is localized mainly in the neck, midpiece, and tail regions of human sperm, is increased during sperm capacitation; however, Hsp90 localization does not change in sperm in response to Percoll isolation or during capacitation. Biggiogera et al. previously reported that Hsp90 is detected only in the cytoplasm of sperm during the elongation stage of mouse spermatogenesis, and is virtually undetectable in late spermatids around the time of spermiation and in mature sperm [34]. Notably, their data suggest that the presence of Hsp90 in the cytoplasm of the sperm head is a marker of mouse sperm immaturity. Similarly, we speculate that the few sperm with Hsp90 expression in their head regions in this study are immature. Therefore, Hsp90 expression may be used as an indicator of sperm quality and function. Wu et al. reported that Hsp90 is localized mainly in the cytoplasm of stage VII–VIII spermatids and in the spermatogonia of rabbit testes [35], but is not detected in mature sperm. HSPCA (Hsp90) is detected within the equatorial region of the head of human sperm [36]. The reasons underlying these differences in Hsp90 expression are not known, but may be explained by species differences or different developmental phases in the testis, epididymis, and ejaculate. These differences could also possibly arise because of the different sources of the antibodies used to detect Hsp90. Our results are in agreement with the recent finding that Hsp90 is present in the neck and midpiece of both boar and cat sperm, as well as throughout the tail of dog and stallion sperm [37]. In the present study, we showed that Hsp90 is localized in the neck, midpiece, and tail regions of human sperm, the regions which drive sperm motility; therefore, this localization pattern is consistent with Hsp90 having possible role in sperm motility and hyperactivation.

Both immunofluorescence staining and western blot analysis showed that Hsp90 expression increased over the span of capacitation, indicating that Hsp90 protein translation occurs in human sperm during this process. Previous work showed that the ejaculated sperm of men with oligozoospermia have increased Hsp90 mRNA expression, which is independent of the presence of a varicocele [38]. Our results are in accordance with a growing body of evidence suggesting that protein translation occurs in mature sperm [39], [40]. Ejaculated sperm is capable of using nuclear-encoded mRNAs transcripts for protein translation by using mitochondrial-type ribosomes, while the cytoplasmic translation machinery is inactive [41]. In this study, we showed that Hsp90 localization is primarily in the neck, midpiece, and tail regions of human sperm, regions that coincide with the location in which protein translation takes place in mitochondria. The mechanism controlling Hsp90 synthesis may involve reactive oxygen species (ROS) [42], [43]; ROS are known to increase during capacitation [44]. Increasing ROS stress can induce protein unfolding, which overcomes the functional capacity of Hsp90 [45]. Hsp90 releases the repression of heat shock factor 1 (Hsf1), leading to Hsf1 activation and, subsequently, an increase in HSP90 expression [46].

The increase in Hsp90 expression during capacitation was reduced by Geld treatment in a dose-dependent manner. Transcriptional regulation of Hsp90 involves heat shock transcription factor 1 (Hsf1) [47]. Hsp90 expression in eukaryotic cells is regulated by many factors, and can be mathematically modeled by taking into account the following key components: the inactive and active forms of Hsf1, interaction of Hsf1 with Hsp90, free Hsp90, Hsp90 complexes with unfolded proteins, HSP90 mRNA production, and the degraded Hsp90 and Hsp90 complex [46]. Geld might inhibit Hsp90 expression by specially binding to Hsp90 and inactivating it, leading to dynamic changes in different Hsp90 components. Although Geld activates Hsf1 leading to increased Hsp90 expression [46], this increase did not offset the Hsp90 and Hsp90 complex degradation caused by Geld during capacitation. These results also show that the reduction of Hsp90 expression by Geld is concentration-dependent. Therefore, Hsp90 expression was reduced during capacitation in the presence of Geld. Taken together, these data show that Hsp90 expression is inhibited by Geld treatment.

To the best of our knowledge, we have presented the first evidence showing that Hsp90 plays an important role in [Ca^2+^]_i_ signaling events in human sperm. The [Ca^2+^]_i_ increase required for capacitation is regulated by Ca^2+^ influx via many transporters, such as CatSper channels, and Ca^2+^ released from intracellular stores via various pumps, including Ca^2+^-ATPases [16], [17]. We found that Geld attenuated the rise in [Ca^2+^]_i_ in human sperm during capacitation. This phenomenon can be explained by Geld occupying the ATP binding site of Hsp90, thereby increasing the availability of intracellular ATP [48]. Increased ATP levels accelerate Ca^2+^ efflux via protein kinase C and plasma membrane Ca^2+^ pump isoform 4 [49], resulting in a decline of [Ca^2+^]_i_. The data in the present study are consistent with that of a study showing that Geld lowers intracellular calcium levels [48]. In the presence of P4, [Ca^2+^]_i_ was lower than that in sperm treated with Geld alone. P4 receptor (PR) has been shown to be present in human sperm [50]. Although P4 activates extracellular Ca^2+^ influx through CatSper channels on the plasma membrane [51], [52], Geld may reduce the ability of the PR assembly from binding to P4 [53]. Therefore, these results suggest that Geld disrupts Hsp90 function in affecting [Ca^2+^]_i_ homeostasis during human sperm capacitation.

Geld treatment increased tyrosine phosphorylation of not only Hsp90, but also other proteins expressed in human sperm, suggesting that Hsp90 is involved in the induction of protein tyrosine phosphorylation during sperm capacitation in humans. In a previous study, Hsp90 was found to be tyrosine phosphorylated in human and rat sperm under capacitation conditions, and that tyrosine phosphorylation in mouse sperm was not affected by Geld treatment [13]. However, Hou et al. speculated that Geld promotes nitric oxide production and subsequently augments sperm capacitation in boars [54]. Nevertheless, these discrepant results cannot be excluded by different species. Evidence suggests that calcium negatively regulates the tyrosine phosphorylation cascade associated with sperm capacitation [55]. Decreased [Ca^2+^]_i_ may contribute to the increase of tyrosine phosphorylation that takes place when human sperm are treated by Geld. Thus, the decrease in [Ca^2+^]_i_ is not in conflict with the observed increase in tyrosine phosphorylation. ATP, on the other hand, reportedly upregulates tyrosine phosphorylation [55].

Inhibition of c Src family kinases, the tyrosine kinases responsible for the increase in protein tyrosine phosphorylation that accompanies capacitation, is predicted to upregulate protein phosphatase 2A (PP2A) activity, which consequently dephosphorylates Ser/Thr-phosphorylated substrates [16]. Interestingly, Hsp90 maintains Ser/Thr Akt kinase activity by preventing PP2A-mediated dephosphorylation [56]. Geld-mediated Hsp90 inhibition has been shown to transiently activate Src kinase and promote Src-dependent Akt and Erk activation. Geld treatment also rapidly disrupts the association between Hsp90 and Src, suggesting that Src is activated as a direct result of its dissociation from the chaperone [57]. In addition, Geld inhibits Src–Hsp90 heterocomplex formation and results in an increased rate of Src turnover [58]. Thus, it is plausible that Geld-mediated Hsp90 inhibition increases the levels of protein tyrosine phosphorylation. Regulation of protein tyrosine phosphorylation during capacitation might also depend on the disassociation of Hsp90 complex rather than just the quantity of Hsp90. Therefore, these data do not conflict with the finding that Hsp90 expression is decreased by inhibition by Geld. Together, these results bring new insight into the mechanism by which protein tyrosine phosphorylation increases during sperm capacitation.

We showed that Geld abolishes P4-induced human sperm capacitation, hyperactivation, and motion parameters. These results suggest Hsp90 has roles in human sperm capacitation, hyperactivation, and motility. Geld reportedly inhibits sperm motility in boars [11], but not in mice [13], and Hou et al. showed that Geld promotion of boar sperm capacitation [54]. Our results show that Geld inhibits P4-stimulated sperm function. These discrepant results may reflect species differences. Our data showed that Geld alone has no net effect on sperm function in the absence of P4 stimulation. On the one hand, the increase in Hsp90 expression during capacitation may balance inhibition by low dosage of Geld; on the other hand, increased protein tyrosine phosphorylation promotes capacitation and compensates for the decreased levels of intracellular calcium. P4 was added into sperm suspensions to create conditions that are more similar to natural physiological conditions. When P4 was present, effects of Geld on human sperm function were observed. These results suggest that Hsp90 has a role in human sperm function that is related to the action of P4. P4 regulates sperm mobility parameters and hyperactivation [59] by activating the PI3K–Akt pathway via CatSper [60]. It also promotes sperm capacitation and the AR [20], [25], [29], but through a different signal pathway [60]. As mentioned above, the structure and function of PR is altered by Geld [53], disrupting its interaction with P4. Taken together, these data suggest that Geld-mediated inhibition of Hsp90 affects capacitation, hyperactivation, and motility in human sperm by altering P4 activity.

In conclusion, we show in the present study that Hsp90 is associated with the events involved in regulating human sperm function. Hsp90 localizes mainly in the neck, midpiece, and tail regions of human sperm, and its expression increases during capacitation. Moreover, we show that Hsp90 is involved in intracellular calcium homeostasis, protein tyrosine phosphorylation regulation, and sperm function in response to P4.
